# New Field Suggested by Dr. Williams’s Paper

**Published:** 1897-06

**Authors:** G. A. Mills

**Affiliations:** New York


### NEW FIELD SUGGESTED BY DR. WILLIAMS'S PAPER.

BY DR. G. A. MILLS, NEW YORK.

It was to be expected that the late paper of Dr. Williams would attract more than ordinary attention. Already one editorial says, "We do not see anything new in it." Dr. Williams makes no claims in that direction. What he has done is a demonstration of what others were thinking as true. Another editor has, it is thought, sought to make the paper substantiate his own claims published in his book,—viz., that decay left in a cavity will cause a continuity of decay unless it be powerfully cauterized. In other words, he believes that the pathogenic organisms will keep on in their destructive work. I do not think his claims are intelligently sustained. It has been fully proved in thousands of instances that teeth have been filled over decay and there has been no return of caries, even without the aid of an antiseptic. Yet it cannot be advocated that the use of antiseptics may not be an advantage oftentimes; but my own view is that the environments will be decidedly changed by the cavity being hermetically sealed with a filling, shutting out the organisms absolutely from air and moisture. Dr. Black has emphasized Dr. Williams's paper largely because of the marked ability shown in his work, which has given a definition far exceeding his expectations, and puts the demonstration so clearly before every eye that it would seem that a novice could not fail to see the facts illustrated. To Dr. Black must be accorded great credit for his industry, which of itself is a marvel. Still, it must be recognized that he places more emphasis than is warranted upon his views of mechanical technique. It will doubtless be an aid to a much higher grade of skill in filling teeth, and it is thought that Dr. Crouse's criticisms are timely. A reconstruction of the "interproximate spaces" is, without a doubt, of value. But practical dentistry is of a far-reaching thought, and requires so much and such varied judgment in its application that the best service or the longest cannot be possessed by the largest number in pursuing one routine practice.

I sat and listened to Dr. Williams's statement concerning the usefulness of his demonstrations, and it was emphasized through him that which Dr. Black equally emphasized during his visit to the Stomatological Society a year ago, that the future success of dental practice could not so much be secured on mechanical lines as would come from the consideration of the secretions of the body. This thought I voiced in one of my New York letters to the *Dental Digest*, for it had been much impressed on my mind for a long time that the day would surely come when some one would bring to us knowledge in this direction. Dr. Williams heartily united in the same hope and belief.

In looking at this subject we cannot expect to succeed either on the line of environments or the changing of the secretions.

Some fifteen years ago I read in the *American Dental Journal* an article by some Baltimore man handling this subject of the secretions in quite a masterly manner. I then thought it made a decided impression on my mind. It only needs a hint to suggest that it is a well known fact that the secretions of the body are made very variable by the sudden change of surroundings. Therefore we are face to face daily with these changes, and, I predict, it will be shown sooner or later that there is a decided influence for ill manifested in the mouth, and necessarily the teeth must suffer because of the unfavorable conditions. None of our labor can blot out the cause of disease, but we can do more than we have ever been able to do as yet by a study on these lines, which we are sure that Dr. Williams's paper largely suggests.

